# *Nasa dugo* (‘It’s in the blood’): lay conceptions of hypertension in the Philippines

**DOI:** 10.1136/bmjgh-2020-002295

**Published:** 2020-07-09

**Authors:** Gideon Lasco, Jhaki Mendoza, Alicia Renedo, Maureen L Seguin, Benjamin Palafox, Lia M Palileo-Villanueva, Arianna Maever L Amit, Antonio L Dans, Dina Balabanova, Martin McKee

**Affiliations:** 1 Department of Anthropology, University of the Philippines Diliman, Quezon City, Metro Manila, Philippines; 2 Development Studies Program, Ateneo de Manila University, Quezon City, Philippines; 3 Department of Medicine, University of the Philippines Manila, Manila, Philippines; 4 Department of Social and Environmental Health Research, London School of Hygiene and Tropical Medicine, London, UK; 5 London School of Hygiene and Tropical Medicine, London, UK

**Keywords:** hypertension, qualitative study, health systems

## Abstract

**Introduction:**

Understanding explanatory models is important for hypertension, a leading risk factor for cardiovascular disease and stroke. This article aims to determine what adult patients with hypertension in the Philippines attribute their condition to, how these views might be explained and what the implications are for hypertension management.

**Methods:**

This is a qualitative study drawing on 71 semistructured interviews (40 initial and 31 follow-up) and four focus group discussions with patients diagnosed with hypertension. The setting was urban and rural low-income communities in the Philippines.

**Results:**

Four prominent perceived causes were identified—genetics, heat, stress and diet—for what patients refer to as ‘high blood’. We propose a ‘folk physiology’ that rests on local understandings of blood and blood flow, draws from broader cultural notions of illness causation and accounts for a dynamic, non-chronic view of hypertension that in turn informs the health behaviours of those affected.

**Conclusions:**

By understanding that hypertension is frequently seen not as a chronic constant condition but rather as an episodic one triggered by external influences, although in those genetically predisposed to it, it may be possible to address patient’s beliefs and thus adherence to treatment.

**Summary box**

**What is already known?**

- Cultural knowledge and local perceptions about particular illnesses can affect health seeking behaviour and clinical encounters.

**What are the new findings?**

- We present an explanatory model for how the causes of hypertension are understood in the Philippines, with ideas—genetics, heat, stress and diet—all drawn from notions of blood.

**What do the new findings imply?**

- Our findings can explain Filipino patients’ self-care practices, as well as their view of hypertension as a dynamic, non-chronic state, both of which have consequences for hypertension management.
- They also illustrate how broader cultural theories can influence the way a specific medical condition is conceptualised and experienced.

## Background

Cultural beliefs structure how people make sense of health and illness, forming ‘explanatory models’ which ‘offer explanations of sickness and treatment to guide choices among available therapies and therapists and to cast personal and social meaning on the experience of sickness’.^1^ Understanding these models is especially important for hypertension, a leading risk factor for cardiovascular disease and stroke.^2^ Despite existence of safe, cheap and effective treatment, lay perceptions of hypertension can be a barrier to control^3 4^ through poor adherence to treatment.^5–8^ Such an understanding can support adherence-promoting interventions that move beyond the knowledge deficit model (ie, that poor adherence is simply due to poor health literacy).^4^

Recent health reforms in the Philippines—including increased revenue from tobacco taxes— have increased access to free medications and improved patient care.^9^ Recognising the growing burden of non-communicable diseases, now affecting one in four Filipinos, with higher rates in urban areas,^10–12^ the Department of Health is promoting health education and provision of antihypertensive medications. However, despite health promotion campaigns that have focused on modifiable risk factors such as tobacco and alcohol use, diet and exercise,^13^ hypertension prevention and control remain a challenge, especially among low-income patients^14^ with intention, knowledge and healthcare system factors serving as barriers to care.^15 16^

Most scholarly attention to hypertension in the Philippines has focused on biomedical aspects of treatment, epidemiology and clinical outcomes, largely ignoring its social and cultural dimensions. Rueda-Baclig and Florencio^17^ provide a rare exception, conducting focus group discussions (FGDs) among young adults without any history of hypertension to elicit their understanding of the disease, documenting several perceived causes, including food, lifestyle-related factors, environment and physical activity. Although such studies make important contributions to the evidence base, ethnomedicinal studies that explore underlying lay concepts of health and illness that inform patient behaviours are lacking.^18 19^

Here we contribute to this literature by identifying ways that Filipino patients with hypertension, particularly in low-income families, conceptualise their condition. We analyse their explanatory models to propose a ‘folk physiology’^20^ that can explain their perceptions of causes of hypertension, drawing from qualitative data (semistructured interviews and FGDs). This is especially important given the growing burden of hypertension in the Philippines,^10^ where cardiovascular diseases (CVDs) now constitute the leading cause of death.^21^ Understanding sociocultural factors that inform people’s attitudes to their hypertension and treatment behaviour can guide clinicians’ interactions with patients along different stages of the care pathway and help policymakers craft communications strategies for health promotion. Moreover, it can contribute to the scholarly literature that focuses on lay conceptions of illness as forms of social knowledge in their own right that play a key role in framing illness and treatment experiences—many of which do not fit biomedical understandings of ‘disease’.^22^ While various theories have been proposed to explain how people make sense of illness in the Philippines and in the wider region—for instance, the humoural theory and the hot–cold theory of disease^20 23 24^—exactly how these diffuse forms of knowledge are mobilised locally, and how they relate to particular biomedical conditions like hypertension, remain unclear.

## Methods

This paper adheres to the Standards for Reporting Qualitative Research reporting guidelines.^25^ We draw on qualitative data gathered in the mixed-method longitudinal Responsive and Equitable Health Systems—Partnership on Non-Communicable Diseases (RESPOND) project which examines patient pathways and barriers to hypertension care in the Philippines and Malaysia.^26^ The Filipino qualitative data consist of 71 semistructured interviews with hypertensive patients (40 initial, 31 follow-up after 12 months) and four FGDs with 33 patients aged 35–70 years old in low-income communities in Valenzuela City (urban) and Quezon Province (rural). Those who signified interest in participating in the qualitative study were gathered from a quantitative database of 620. Interview and FGD participants were purposively selected from that grouping according to sex, age and location (ie, urban vs rural) to capture social explanations of hypertension across the life course (table 1). The interviews were conducted in Filipino (Tagalog) by the first and second authors and typically lasted from 45 to 60 min. Interviews were audio recorded, transcribed verbatim and translated into English. The interviews elicited patients’ illness narratives and views on hypertension, as well as their experiences with the healthcare system. Emergent themes in initial interviews were further explored during follow-up interviews and FGDs. The FGDs lasted from 75 to 90 min and focused on lay concepts on hypertension, patient pathways and barriers to care.

**Table 1:** Characteristics of interview participants

Age group	Urban		Rural		Previous hypertension diagnosis	
	Female	Male	Female	Male	Yes	No
30–49	7	1	3	2	11	2
50–70	6	6	10	5	22	5

Those in the rural areas are mainly involved in agriculture (eg, farming and fishing). Those in the urban areas are mostly informal and contractual workers (eg, drivers, vendors, house help and factory workers). The study included participants who did not receive any previous hypertension diagnosis but were measured to have elevated blood pressure by the RESPOND quantitative team.

RESPOND, Responsive and Equitable Health Systems—Partnership on Non-Communicable Diseases.

Our analysis, which was primarily conducted with the original Filipino transcripts by the first and second authors (GL and JM), was guided by principles of grounded theory.^27 28^ We followed an open-coding approach (facilitated by NVivo) to ensure lay theories emerged inductively from the data, illuminating patients’ explanatory models and perceived causes of illness. Prominent recurrent themes were identified through consensus between the first two authors, paying close attention to illness semantics.^29^ Themes from the initial interviews were further explored and refined via FGDs and follow-up interviews, and via discussions with the wider research team, involving both local and foreign researchers. Reflexivity was considered throughout data gathering and analysis, in recognition of the inherent subjectivity of researchers and study participants.^30^ During an interview, the interviewer and interviewee each react to the characteristics and perceived position of the other, which contributes to the coconstruction of reality yielded through the interview process.^31^ The usage of grounded theory enabled us to avoid being limited by preconceptions about hypertension, given that many of the research team have medical backgrounds and have experienced patients’ knowledge and understanding in clinical settings. Moreover, interviews were conducted by the first two authors who shared the participants’ language, thereby mitigating (although not eliminating) power dynamics which may arise when interviews are conducted by a cultural ‘outsider’ facilitated through a translator.

### Patient and public involvement

The mixed-method RESPOND project, within which the current paper is situated, actively engaged with stakeholders and community representatives including patient groups, during the project inception phase to identify major challenges faced by poor and hard-to-reach populations, along with cultural considerations which may impact the study.^26^ This engagement was maintained and strengthened throughout the field work phase of the project.

Individual participants are identified using pseudonyms.

## Findings

Blood emerged as the bodily element through which ‘high blood’ (which is how patients refer to hypertension) is *experienced* and made a reality for patients, and changes in the blood were seen as the mechanism through which hypertension is understood to operate in the body. In other words, blood is central to the ‘folk physiology’ of hypertension for many of our participants.

The centrality of blood is evidenced by the participants’ illness semantics. Aside from the term ‘high blood’, its literal Tagalog translation ‘mataas ang dugo’ or ‘malakas ang dugo’ (strong blood’) were also used to refer to hypertension. It was seen conceptually as one pole on a spectrum of blood states—the opposite of which is ‘low blood’ or its literal equivalent ‘mababa ang dugo’ (which conflates hypotension or low blood pressure and anaemia). Low blood features symptoms like dizziness and weakness, as Lorena, a-44-year-old female driver employed in a school transport services from Valenzuela described:

Aside from high blood, I also had low blood…my blood pressure was low and I was always dizzy. So I ate *talbos ng kamote* (a leafy vegetable with a red dye), and the low blood only lasted for a while.

The centrality of blood is also reflected in the way ‘high blood’ was understood to affect the body. Hypertension exists for many of the participants because of changes in the blood, particularly, in its perceived *viscosity* and *temperature*. In terms of viscosity, blood can either be *malabnaw* (thin or watery) or *malapot* (thick or viscous), with the latter state being associated with ‘high blood’ and the former with ‘low blood’. ‘Thick blood’ can lead to various symptoms, like headache and a thickening of the neck. In the long term, thick blood is also viewed to cause clogging of the blood vessels, as Fernando, a 64-year-old farmer from Quezon, explains, ‘the blood goes up because of the *litid* (vessels) are *nababara* (clogged) so the blood cannot flow.’ In terms of temperature, blood that is *mainit* (hot) or *kumukulo* (boiling) is likewise linked to high blood pressure—as will be discussed in the ensuing presentation of findings.

Blood and changes in blood were central to four main, overlapping causes for hypertension identified by study participants—all fairly similar in terms of frequency of mentions:

- *Namamana* (‘Inherited’)—The predisposition for having hypertension is inherited, and that this genetic component is ‘nasa dugo’ (in the blood).
- *Init* (Heat)—Hot weather or init (heat) can trigger episodes of hypertension by causing blood to heat up or boil.
- ‘Stress’—Physical and emotional stressors can lead to hypertension likewise by causing blood to heat up.
- *Pagkain* (Diet)—Particular foods (and beverages) cause hypertension by making blood thicker and more *malambot* (viscous).

**Figure 1:**
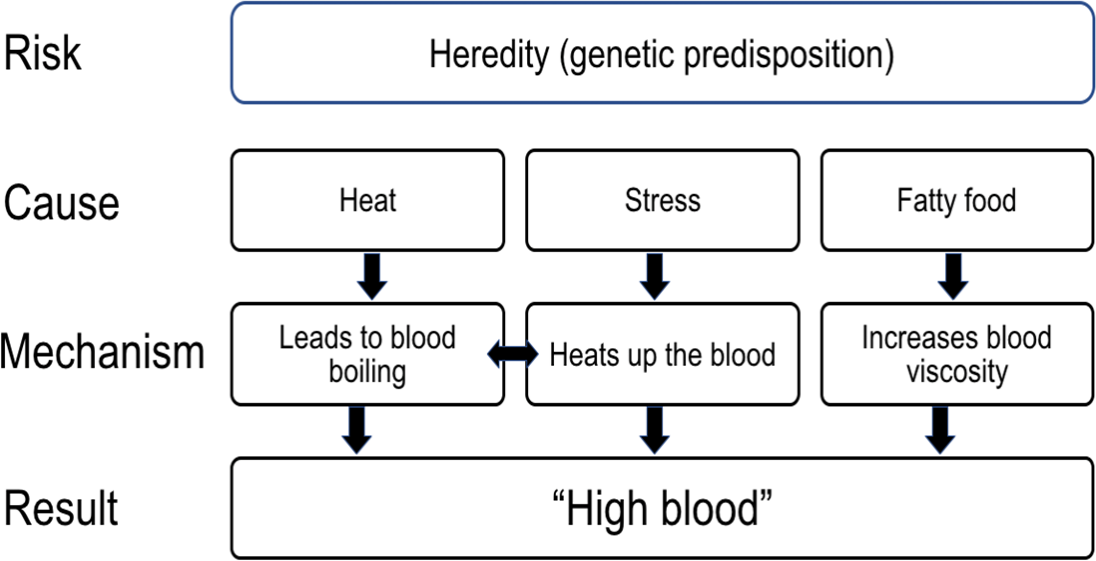
Folk physiology of hypertension.

We now discuss each of these perceived causes, which are summarised, and their relationships illustrated, in figure 1.

### ‘Namamana’: folk genetics

Many study participants viewed hypertension as ‘namamana’ or inherited through blood. Some viewed deaths among family members due to stroke or ‘atake sa puso’ (heart attack) as meaning that they could suffer the same fate. This notion of genetic risk is evidenced by discourses around adherence to treatment and self-management.

Delilah, 42, female, a homemaker with a small livestock business in Quezon, provides an illustrative example. On the one hand, ‘she wasn’t shocked’ about having been diagnosed with hypertension because she knew the disease is in ‘in her blood’—but she was also apprehensive: ‘I was scared since my mother, father and other siblings had stroke. I don’t want that to happen to me as well’. Ultimately, she talked about how the hereditary nature of hypertension was a motivation to access health services:

Interviewer: What made you decide to get a treatment during that time?

Delilah: Since it runs in our family, I learned how to take BP and when the time came when I was getting high blood pressure results, I decided to go to the doctor to get professional advice.

This view is shared by Linda, 48, female, a community health worker in Valenzuela:

My mother already had high blood. And on my mother’s side…others (had a similar condition). I really thought about it. I said to myself ‘I don’t want that to happen to me, when I am older’.

These comments point to a perception of inherited predisposition to hypertension. There is also an underlying notion of this susceptibility being mediated by the blood. As Kristina, a housewife from Quezon, mentioned: *high blood that is inherited is natural to the body, it’s unavoidable, it just arrives.* It also points to an understanding of people having different types of blood depending in part with their family, which can influence the seriousness they attach to hypertension prevention, diagnosis, treatment and self-care.

‘It’s because of the heat’: Hypertension and temperature

Heat is also perceived as a trigger of hypertension—or more precisely, hypertensive episodes, especially among urban participants. As Ernesto (60, male, retired factory worker from Valenzuela) shared, this perception is also linked to changes in blood:

I don't know but maybe it’s because of the climate because it’s really so hot. It’s not like we’re living in an air-conditioned house. If it’s hot, your head also heats up, your blood boils, and so when they get your B.P., of course it’s high.

For her part, Rosita (58, female, a homemaker from Valenzuela) lists heat among three perceived causes, alongside exhaustion and fatty foods. As the conversation below shows, heat is seen as a risk factor, taken seriously to a point that it informs certain practices—although likely for several reasons other than fear of hypertension:

Rosita: Sometimes my BP goes down but because of fatigue and the heat, and it goes up again.

Interviewer: Is it really hot around here?

R: Very much… […] That’s why when we sleep at night all our windows are open.

I: That’s why you have a lot of electric fans here due to the heat.

R: Very much. It can kill you.

Views of heat as a trigger of hypertension can shape people’s behaviour, including self-management, with participants using fans or avoiding heat to try ‘lower’ their hypertension or maintain health. These ideas were apparent during fieldwork in the way participants organised space; multiple electric fans were commonly used during interviews and referred to by our participants as a way of addressing changes in hypertension status, in addition to their immediate function of alleviating discomfort, particularly in urban areas where the humidity and daytime highs of 32°C–35°C can be become overbearing in their small, tightly packed and poorly ventilated houses.

These temperature-related ‘care’ practices were adapted to work within the constraints of people’s livelihood and everyday work, as Cora, 62, a female farmer from Quezon Province shared:

Lucia, my friend, told me not to stay long under the sun because my (blood) pressure might go up. My job is farming so I’m always under the sun. If I cannot bear the heat, I go under a shade. I also wear a hat as a protection from heat.

The link between heat and hypertension supports participants’ view of blood as a dynamic state that can ‘boil’—but can also cool down, depending on the weather (as in the case of Ernesto above). This is also illustrated by Nerissa (female, a 54 year-old homemaker), a respondent from Quezon who owns a *sari-sari* store (neighbourhood store) outside her house:

Interviewer: How can you say you have high blood?

Nerissa: I feel dizzy especially when it’s hot. I get high blood for sure. But it subsides after a while.

Aside from signifying discomfort with high temperatures and humidity, heat also serves as a metaphor for anger and similar emotional states, as will be discussed in the following section.

### Stress

Stress (which is also word commonly used in Tagalog) also figured in people’s perceived causes of particular episodes of hypertension and in their views on the type of self-management practices that would help address or manage it. These figurations of stress (both physical and emotional) are often linked to both heat and changes in blood, likely in both the metaphorical and experiential sense. As Ernesto said:

When you’re irritated, especially when it’s hot, your head will heat up. And your body will be affected. Your blood will boil. Then when they take your B.P., as expected going to be it’s high.

Barbara, 62, a female community health worker from Valenzeula, echoed the same sentiments, speaking of the following preventive measures:

If your blood is high, you should avoid thinking or getting angry…. You shouldn’t think too much, it will make you tired, iinit ang ulo mo (literally: your head will heat up)

While Barbara emphasised emotional stress, Mariano (64, male, farmer) from rural Quezon, highlighted physical stress:

The tiredness as well (can lead to high blood). For instance, you had trip, you travel, you walk, then you work again. Obviously, your blood pressure will increase. But when you take a rest first, it will not, maybe. It will just be neutral.

However, not all strenuous activity is associated with stress leading to ‘high blood’; physical exercise was recognised as beneficial when done as a recreational activity—as opposed to work-related physical effort, further highlighting the role of emotions in this explanation. As this exchange between focus group participants in Valenzuela illustrates, this ‘healthy’ form of physical exertion is also articulated in terms of blood flow:

Participant 01: You have to move your body for the blood to flow. Exercise is a good way of getting tired. It’s healthy for the circulation because it releases toxins from the body.

Facilitator: But what about doing household chores?

Participant 02: That can be tiresome and stressful because you are doing and thinking at the same time for the whole day. It’s like a bad kind of exercise that can make you high blood. (laugher among the participants)

### Diet

Most of the study participants say that their diet has contributed to their hypertension, particularly, their intake of foods they consider ‘unhealthy’ such as those that are *maalat* (salty) and *mataba* (fatty). Blood, once again, figures as the medium through which food exerts its effects on hypertension: participants speak of eating too much food, particularly those perceived to be high in cholesterol, as causing blood to be more *malapot* (viscous) and thus cause *bara* (clogs) in the blood, which, in turn leads to ‘high blood’ and the complications they attribute to it: stroke and heart attack. Cholesterol figures prominently in their accounts, but it must be noted that the local notion of ‘cholesterol’—understood simply as a substance that comes from *taba* (fat)—is not the same as its use in biomedicine.

This conversation with Mariano is illustrative of how cholesterol figures in their explanatory models:

Ah, eating foods with so much oils, people who likes fried food, meat (increases cholesterol).[…]I am guilty (of eating those foods) because I live alone. Because whenever I go farming in up in the mountains, it is easy to cook a whole chicken and I do not know that chicken has high cholesterol. It has cholesterol but I do not know if it’s high or not. I will just pack it and what I eat in the morning, I bring it in mountain. I consume all of it, obviously, my cholesterol will increase.

Particular notions of ‘cholesterol’ emerge from these accounts. For instance, cheap foods are seen by the participants as having higher cholesterol while their expensive ones are seen as healthier. This is highlighted in a conversation Aida, female, a 45-year-old homemaker and businesswoman from Valenzuela:

Consume only two eggs per day and it must be boiled. If you’ll eat fried egg, it has oil. You should only use olive oil. It does not contain cholesterol, right? It’s free of cholesterol, however, it’s expensive.

Another notion is that specific foods are particularly high in cholesterol—like crab and pork skin. In one of the research sites—a coastal community where crabs are affordable to low income households—one participant narrates:

If I eat crab, I start to feel that pain on the back of my neck. I avoid it because we must have a self-discipline and we are the only ones responsible for our treatment. So it’s up to us. You should just eat small portion and not large portion. All excess is not good even if you’re allowed to eat it

In the above, we see the immediacy of the food’s relationship to ‘high blood’; the fattiness or the perceived ‘cholesterol’ translates to symptomatology; it is perceived to have an immediate effect on the body. Conversely, the inability to eat adequately is linked to ‘low blood’.

As synthesised from various interviews and FGDs, some foods were also seen to make the blood *malabnaw* (thin)—and therefore be beneficial to hypertension—including fruits that are *maasim* (sour)—like citrus and green mango, as well as vegetables. On the other hand, foods like *balut* (fertilised duck egg), crabs, fatty meats (eg, roasted pork), *gata* (coconut milk) and alcoholic beverages such as beer were seen as *malapot* and are seen as beneficial to ‘low blood’ or anaemia. One generalisation gleaned from the food items mentioned is that sour foods and beverages appear to be linked to *thinning* the blood and lowering blood pressure, while those that are sweet and fatty are perceived to do the opposite.

These local notions also influence their self-care practices, as seen in this discussion with Pacito, a 39-year-old fisherman from Quezon:

Pacito*: As what people say here, when you have high blood, you have malapot na dugo (viscous blood)*.

Interviewer: What happens when you have malapot na dugo [viscous blood]?

P: When you eat too much fatty and oily foods that can get stuck in your body and (can have bad effect to) your blood circulation. That’s why I stopped my maintenance (regular medicine for hypertension) and I drink kalamansi juice because they say that can make your blood thinner and I’ve mentioned before that I think my medication has side-effects to my throat. I didn’t like it. Sometimes when I’m fishing, we bring food there and condiments. I drink vinegar if it happens I get high blood out at sea.

## Discussion

### Explanatory models

Our findings complement and add to the limited existing literature on popular explanatory models of hypertension in the Philippines. For instance, while not elaborating on its significance, Rueda-Baclig and Florencio mentioned ‘viscosity of the blood’,^17^ and recognised the centrality of blood ‘flow’ in conceptions of hypertension. Notably, they also reported how some respondents viewed hypertension and anaemia as opposite conditions, of ‘high blood’ and ‘low blood’. Our study illustrates how these blood-based mechanisms create a dynamic non-chronic view of hypertension that draw from both local knowledge and biomedical ideas. Reviewing ethnographic and cultural historical literature in the Philippines, medical anthropologist Michael Tan affirmed the role of blood in ‘folk genetics, reinforcing the ‘nasa dugo’ (in the blood) explanation from our participants.^20^ Tan also draws a link between heat and blood that bears striking parallels with the accounts we elicited.^20^

Blood is believed to thicken when ‘overexposed to heat or cold’, causing illnesses like high blood pressure and heart illness because its viscosity results in sluggish circulation as well as a sticking to the walls of the veins. This folk theory of blood viscosity actually comes quite close to the ‘modern’ explanation.

Another review of this literature reveals connections that participants in our study did not explicitly articulate, but that might help explain their views of which foods are beneficial for (and detrimental to) hypertension. Participants’ views align with the categorisation of food as either ‘hot’ or ‘cold’ in Filipino folk medicine, where meats belong to the former and most fruits and vegetables to the latter.^32^ These local food taxonomies serve as a link between two of the causes identified above—heat and diet.

Beyond the Philippines, our findings provide interesting comparisons with the global literature. For instance, a systematic review of lay perspectives on hypertension and drug adherence^4^ found that stress, food, being overweight, family history and lack of exercise were the most common perceived causes of hypertension. Heat was only identified as an aetiological factor in Brazil, Thailand, Israel and the USA. Notably, the notion of ‘thick blood’ was also reported in one paper among African-American women in New Orleans.^33^ A belief that hypertension was due to excess blood in the body was reported in Ghana^34^ while stress was invoked in a Kenyan study.^35^ Meanwhile, a mixed-methods observational study of seven Asian countries (including the Philippines) found that patients ‘were not concerned with reaching any target blood pressure goals and accepted fluctuations in blood pressure’.^36^

The broader explanatory models that underline these attitudes need to be explored further: as others^20 23^ have documented, there are similar concepts in folk medicine in the region and elsewhere in the world.^37–39^ For instance, humoural theory and the related ‘hot–cold syndrome’, elements of which have been documented in the Philippines, may inform the emphasis on blood, heat and balance that figure prominently in our participants’ narratives—and underwrite notions of balance and flow that are inherent in them and could inform their overall approach to health.^16^

The existence of these ‘grand’ explanatory models, however, does not account for the ways in which participants relate pressure and viscosity, and their incorporation of biomedical notions such as ‘cholesterol’. This suggests that they are rooted in long-running beliefs but explanatory models are iterative, incorporating biomedical concepts and semantics. The challenge, then, is to document not just explanatory models but how they evolve over time and across sociocultural contexts.

### Implications for policy and practice

These findings are important for policy and practice. As noted above, they can inform the design of interventions that are tailored to local circumstances. However, by incorporating the lessons into clinical practice, this knowledge can promote a shared understanding between health professionals, who may come from quite different sociocultural backgrounds, and their patients. In this case, a blood-based folk physiology can help explain the ‘dynamic’ or ‘non-chronic’ view of hypertension and high blood pressure, as a condition that comes and goes, or, in the words of one participant, as an ‘on and off’ illness. While one of the above causes—genetics—suggests a deterministic notion of hypertension, participants frequently talked about moving from states of ‘high blood’ to ‘low blood’ depending on practices one engages with (eg, eating fatty food). The illness semantics are also illustrative, with many participants saying ‘Na-hi-highblood ako’ (‘I am having high blood’) as opposed to ‘May high blood ako’ (‘I have high blood’). This dynamic view can help make sense of low adherence rates in the Philippines and the region^17 36^—even as lay and biomedical ideas are not necessarily contradictory. For instance, the dietary principles volunteered by our study participants generally align with biomedical nutritional advice, while emotional and occupational stress has been associated with the development of hypertension.^40^ As Tan^20^ notes, ‘The challenge, then, with biomedical validation, is to bring together the measurable conditions and changes with the world of symbols and meanings that people have, and to understand the social context in which all this is created and recreated’.

The blood model can also help explain how people experience and make sense of symptoms. Thus, some participants speak of symptoms like ‘headache’ and ‘thickening of the neck’ as the consequence of the thickening of the blood. Because our participants think that they can *feel* their blood, they also think that they can feel ‘high blood’; for them, a sphygmomanometer often serves to confirm—not detect—hypertension. This *legibility* of hypertension in terms of ‘folk physiology’ can explain the sporadic nature not just of medication-taking but also of BP monitoring. It can also lead to a failure to act on, and seek care for, hypertensive emergencies—as very high blood pressures in the presence of only mild symptoms will not be regarded as such. The promotion of BP monitoring, then, must necessarily involve reminding patients that blood pressure and symptomatology are not always related.

The findings of a blood-based folk physiology can illuminate the ways in which participants relate different diseases to each other. Like Rueda-Baclig and Florencio,^17^ we found that some respondents viewed hypertension and anaemia as two opposite conditions, raising the possibility that they see the two conditions as mutually exclusive: a view consistent with local notions of balance.^20 41^ Thus, patients prescribed medications for both anaemia and hypertension may find it absurd, leading to non-adherence. Tan’s observation that ‘people with insomnia, anaemia, and generally weak body resistance (*sakitin*, or sickly), are said to have ‘thin’ blood’^20^ suggests that there are more illnesses that people interpret using this folk physiology, serving as an impetus for research beyond hypertension.

These findings also reinforce the rationale for health promotion campaigns to work with communities in a participatory way,^42–44^ recognising (and anticipating) their local conceptions rather than simply seeking to displace them by the transfer of biomedical knowledge, and acknowledging how some of their self-care practices (eg, choosing foods that can make the blood ‘thinner’) can be mobilised for shared therapeutic goals.^45^ This is important if we are to encourage ‘critical consciousness’^46^ about how some folk ideas might enable or undermine agency to prevent and manage hypertension in their everyday life and is an important first step towards empowering people to take control over their health. Importantly, this participatory health promotion work must also enable communities to critically reflect and act on the ways in which biomedicine ‘constructs’ the body and marginalises local vocabularies with which to articulate illness and suffering.^47^

Finally, these findings point to directions for future research. Given the centrality of blood in people’s conceptions of illness, further work documenting and elaborating related beliefs can be useful both for hypertension and for other medical conditions: a project that necessarily involves how people relate these conditions (eg, hypertension and anaemia as being ‘high blood’ and ‘low blood’, respectively), how metaphors such as ‘heat’ are embodied in people’s illness experiences, and, conversely, how corporeal experiences give rise to such metaphors.^48^ This is particularly true for the Philippines but may be relevant for other countries, where concerns about blood may also inform people’s attitudes.

## Conclusion

This paper identified perceived causes of hypertension among these poor communities in the Philippines, related to genetics, heat, stress and diet. These are perceived, in a cultural explanatory model, as acting by changing the characteristics and behaviour of blood. Collectively, they contribute to a folk physiology of hypertension that those in these communities use to explain the aetiology of the condition and influence non-medical forms of self-care. This folk physiology also accounts for a view of hypertension as a dynamic, non-chronic condition.

The study has certain limitations, which future research can address. Our findings likely reflect our exclusive scope on low-income areas, potentially limiting applicability to middle-income and high-income Filipinos. Its focus on hypertension, moreover, precludes the relevance of the centrality of blood to other conditions hinted at by our participants. Furthermore, characteristics of the researchers (eg, socioeconomic differences) and methodological constraints (eg, interviews vs longer-term fieldwork) could have affected what the participants shared with us.

These limitations notwithstanding, the study shows how local knowledge, shared by rural and urban communities, shapes the way a specific medical condition is understood and acted on, and how these notions and practices can then inform preventive and care practices. The way that hypertension is frequently seen not as a chronic constant condition but rather as an episodic one triggered by external influences is a particularly relevant for efforts to address adherence to treatment.
